# The LoCo (Lockdown Cohort)-effect: Socioeconomic differences in fertility during the pandemic

**DOI:** 10.1093/eurpub/ckac131.095

**Published:** 2022-10-25

**Authors:** M Oberndorfer, AM Tilstra, R Dundas, AH Leyland, A Pearce

**Affiliations:** 1 Center for Public Health, Medical University of Vienna, Vienna, Austria; 2 MRC/CSO SPHSU, University of Glasgow, Glasgow, UK; 3 Leverhulme Centre for Demographic Science, University of Oxford, Oxford, UK

## Abstract

**Background:**

The economic and social disruptions caused by the COVID-19 pandemic and its mitigation measures may have affected fertility unequally across social strata. If a compositional change in maternal socioeconomic characteristics is confirmed, counterintuitive changes in future population health - the LoCo-effect - are likely.

**Methods:**

We analysed data from maternal inpatient discharge records containing births between January 2018 and November 2021 by Scottish Index of Multiple Deprivation (SIMD) quintile. We used monthly number of births before November 2020 to estimate expected monthly births after November 2020 and compared against observed births in each SIMD quintile. Further, we estimated associations between monthly average stringency of national mitigation measures (Stringency Index (0-100)) and births 9 to 13 months later using distributed lag models.

**Results:**

Between November 2020 and November 2021, there were 1301 (10.3%) fewer births than expected for the most deprived quintile (Q1; 953 (8.7%) and 375 (4.1%) less in Q2 and Q3). In the two least deprived quintiles, however, fertility remained mostly unchanged. A 10-point increase in monthly average Stringency Index in Q1 was associated with an average cumulative decrease of 8.5 births (95%CI: -14.1; -2.8, p = 0.006) 9 to 13 months later. Conversely, this estimate was a 4.4 increase (95%CI: 1.3; 7.5, p = 0.008) in Q5 and a 5.9 increase (95%CI: 1.4; 10.4, p = 0.013) in Q4.

**Conclusions:**

Apart from their exposure to pandemic and lockdowns, it is likely that, due to compositional changes in births, the observed LoCo started life, on average, more socially advantaged than previous birth cohorts in Scotland. We show a substantial decrease in fertility in the most deprived areas, while fertility remained little changed in the least deprived areas. Increases in the stringency of lockdown measures were associated with a decrease in births in the most deprived but an increase in the least deprived areas 9 to 13 months later.

**Key messages:**

• Between November 2020 and November 2021, there were substantial decreases in births among deprived areas in Scotland while fertility in the least deprived areas remained mostly unchanged.

• For these births, the observed compositional shift in maternal socioeconomic characteristics may lead to changes in current and future health and health needs – the LoCo (Lockdown Cohort)-effect.

